# Is obesity associated with decreased health-related quality of life in school-age children?—Results from a survey in Vietnam

**DOI:** 10.3934/publichealth.2018.4.338

**Published:** 2018-09-21

**Authors:** Nguyen Thanh Ha, Do Thi Hanh Trang, Le Thi Thu Ha

**Affiliations:** Hanoi University of Public health, 1A Duc Thang street, North Tu Liem District, Hanoi, Vietnam

**Keywords:** health-related quality of life, obese children, PedsQL 4.0

## Abstract

**Background:**

Overweight and obesity have short-term and long-term effects on children's physical and mental health. These conditions currently have a tendency to increase among Vietnamese school children.

**Aims:**

This study aimed to assess the quality of life among 8–10 year-old children in Vietnam.

**Methods:**

This cross-sectional study was conducted with a sample of 264 children aged 8–10 years (including 88 obese children and 176 normal-weight ones) at two primary schools in Hanoi, Vietnam, in 2018 and their mothers or fathers participated in this study. The Vietnamese version of the Pediatric Quality of Life Inventory (PedsQL) 4.0 generic score scales were used to collect information from children and their parents about the children's quality-of-life. Mean and standard deviation of PedsQL scores were calculated. Independent t-test was used to compare mean scores between normal-weight and obese children.

**Results:**

Both child self reports and parent-proxy reports revealed that obese children had significantly lower scores for the total scales compared to normal weight children (80.7 versus 84.0; *p* < 0.05 for proxy reports and 77.6 vs 84.6; *p* < 0.001 for self reports). Total scale and subscale scores reported by parents were lower compared to those reported by children. Besides, 79% of normal weight children reported having PedsQL total scores in the highest quartile (≥75%), compared to 56.8 % of obese children (*p* = 0.01). Similarly, these proportions for parent-proxy reports were 79.5% and 65.9%, respectively (*p* < 0.05). Emotional scores were both found the lowest among the four subscales (71.6 for child self reports and 73.1 for parent-proxy reports).

**Recommendations:**

Interventions aimed to improve quality of life of overweight and obese children in Vietnam should not focus only on diet adjustment and physical exercise but need to address all dimensions of health-related quality of life, especially emotional, social and school functioning.

## 1. Introduction

Overweight and obesity in children has been on an alarming increase, at a rate of 10% per annum [1]. The WHO reported that 41 million under-5 children suffered from overweight and obesity in 2014 [2]. By 2020, the prevalence of overweight and obesity in children worldwide are expected to be 9.1%, equivalent to 60 million children [1]. In Vietnam, obesity has been on a rapid rise and become a public health concern, especially in big cities. In Hanoi, the capital city of Vietnam, the prevalence of overweight and obesity among the 8–11 year-olds in 2003 was 7.5%, increasing to 12.9% in 2009 and 39.3% in 2013, of which the obesity rate was 11.2% [3]. In Hochiminh, the second largest city in the South of Vietnam, a survey on elementary school students in school year 2002–2003 showed that the overweight and obesity prevalence was 9.4%, but in school year of 2008–2009, obese students formed 7.7% at some schools, and this figure rose to 38.5% in 2012 among the 6–11 year-olds [4]. Overweight and obesity have resulted in serious physical health problems; they increases the risk of cardiovascular diseases, hypertension (or high blood pressure, HBP), and metabolic or endocrine complications a systematic review by Buttitta and colleagues shows that overweight and obesity affect not only children's physical health but also their mental health. Overweight and obese children have higher risk for symptoms of depression and anxiety, lack of confidence and social stigma. Psychological complications have negative effects on their quality of life [5]. Overweight and obesity have detrimental effects on children's health and school functioning, leads to stigmatization and discrimination, weight-based teasing and bullying, resulting in various psychological and mental consequences.

A large body of research in quality of life (QOL) has been conducted in different parts of the world, using different health-related quality of life measures. Based on the WHO's definition of an individual's health, the Pediatric Quality of Life Inventory (PedsQL4.0) was developed in the United States of America to assess the QOL of children. Early dection of children's QOL allows us to have an overview of the effects of obesity on their physical, mental, social and school functioning in order to conduct timely interventions and minimize the impacts of obesityon children. Strengths of the PedsQL include brevity, convenience for use in both clinical and community settings and availability of parallel reports by the child and his/her parent. In addition, the tool has been reported as having satisfactory reliability and validity and having relatively low ceiling effects, which occur if most participants achieve a near perfect score on a questionnaire [6],[7].

A large number of studies worldwide, have used the PedsQL to assess QOL of obese children [8] and consistently indicated that generally, obese children had lower QOL scores in terms of physical, emotional, social and school functioning than their normal weight peers [9]–[15]. For example, according to Schwimmer et al. obese children and adolescents on weight-loss treatment had poorer QOL than their normal weight peers in all aspects (physical, emotional, social and school functioning), but their QOL score was similar to that of children and adolescents diagnosed with cancer [13]. Similarly, a study of Australian children pointed out an association between lower QOL and obesity [14], and another clinical research of obese children with sleep disorders also found a low QOL score among this sample [15]. There has also been evidence of an association between obesity and different aspects of QOL. For instance, Chinese obese children were 2–5 times more likely to face problems related to physical, social and school functioning than those with normal weight, and overweight ones were at risk of encountering social functioning-related problems twice as much as their normal weight peers [10].

In Vietnam, recent research has focused on assessing the prevalence and risk factors of overweight and obesity as well as interventions that aim to reduce the prevalence of overweight and obesity, but no literature documenting health-related QOL of Vietnamese overweight and obese children in a comprehensive way is currently available. Additionally, no studies have compared parents' and children's perspectives of how health varies according to weight and none have been performed in community samples of elementary school children. The parents' perspective about the child's health in particular and health-related quality of life in general is important because it can be a strong driver of health service use. This paper, using data from a sample of Vietnamese school-aged children, aims to explore health-related QOL of obese children in comparison with normal weight children reported by themselves and their parents. The study would inform policies and prevention programs that aim to prevent obesity and its consequences among Vietnamese children, which are currently focusing only on changing dietery patterns and promoting physical acivities. Specifically, the study was aimed to provide evidence supporting policies and intervention programs that comprehensively involve not only promoting children's physical health but also strengthening their psychological wellbeing and school and social functioning.

## 2. Materials and method

### 2.1. Study design

This cross-sectional study was conducted in several stages between June 2017 and March 2018. Step 1: Measuring children's height and weight to classify them into two groups: normal weight group and obese group. Step 2: Using a validated instrument—the Vietnamese version PedsQL 4.0 generic score scales (child self-report scale and parent-report scale)—to assess the QOL of overweight and obese children aged 8–10 years. The Vietnamese version of the PedsQL 4.0 generic score scales (both child-self report and proxy-report) have been validated and results of the validation study are available upon request.

### 2.2. Sampling

The study took place in Hanoi, the capital city of Vietnam. This is the most populated city in the country. Over the past years, the private school system has been growing rapidly in Vietnam; hence, to ensure that the study population represents the Vietnamese child population, the study was conducted in two primary schools in Hanoi, one was a public school and the other was a private school. In order to classify children into two categories of obese and normal-weight, 1,695 students aged 8–10 years at these two schools (grades 3–5) had their height and weight measured. The BMI-for-age Z-score (BAZ) and the WHO 2007 standard were used to assess nutritional status. In this study, 844 children were classified as normal weight (−2SD ≤ BAZ ≤ 1SD), 465 as overweight (1SD ≤ BAZ ≤ 2SD), 348 as obese (BAZ ≥ 2SD) and 38 as underweight (BAZ < −2SD).

The sample size and sampling method for assessing obese children's QOL was as follows. In order to calculate the minimum sample size that hadsufficient power to detect difference (if any) in the mean QOL scores (based on the PedsQL 4.0) between obese children and their normal weight children, we used the sampsi command in STATA version 13.0 with the following parameters: The significance level α = 5%; the power 1−β = 85%; the sample ratio between the obese group and the normal weight-group: r = 1:2; μ_1,_s: The mean QOL score and the standard deviation for the normal weight group; μ_2_, s_2_: The mean QOL score and the standard deviation for the obese group. In Vietnam, as no research had used the PedsQL 4.0 to assess obese children's QOL, we used the mean PedsQL scores and SDs from a study conducted in China [10] as follows: μ_1_ = 75.58, s_1_ = 15.08, μ_2_ = 69.38, s_2_ = 15.01.

Using the above parameters, the sample size for the obese group was 80 children and that for the normal weight group was 160 children. We anticipated a non-response rate of 10%, therefore the final sample size was 264 children, including 88 obese children and 176 normal weight ones. We examined QOL through both child-self report parent-report hence, 264 parents (mother or father depending on their availability) of those children were recruited. The number of obese children selected in each school was proportional to the total number of obese children each school had. The sample of obese children and normal weight children for this study was randomly selected from the list of all obese and normal weight children of the schools. For each child selected, either his/her father or mother (depending on their availability) was also selected to participate in the study.

### 2.3. Data collection

After the nutritional status was identified through anthropometric measurements at the two schools, demographic background and QOL information was collected using anonymous self-administered questionnaires for both children and their parents. Health-related quality of life was measure by an embedded scale, the Vietnamese version of the PedsQL 4.0 generic score scales. The PedsQL 4.0 generic score scales used for children and their parents consisted of the same numbers of items and contents. Twenty-three items of each scale were grouped into four subscales: Physical (8 items), emotional (5 items), social (5 items) and school functioning (5 items).

The detailed information about the study, consent forms and questionnaires were sent to parents to complete at home and return to the research team on a set date. All 264 children and their parents agreed to take part in the study. The children were asked to complete the questionnaire in classrooms under the instruction of the research staff. They were seated far enough from each other to ensure privacy when answering questions (25 children/classroom).

### 2.4. Measures

#### 2.4.1. Outcome variables

Each item of the PedsQL 4.0 generic score scales has 5-levels indicating the frequency the child encountered the problem mentioned in that item, from “never” (0) to “almost always” (4). The raw scores were then converted to a 100-point scale, i.e. 0 = 100, 1 = 75, 2 = 50, 3 = 25 and 4 = 0. Higher scores indicate better QOL. The mean score for each subscale and the total scale were calculated by summing the scores of all items in the subscales and the total scale divided by the number of items answered [18]. QoL scores were examined in quartiles, (≤25, 25− ≤ 50, 50–75 ≥ 75). Scores beloing to the two lowest quartiles indicated low QOL and scores belonging to the two highest quartiles indicate high QOL.

Cronbach's alpha value for each subscale and for the overall scale all exceeded 0.7 for both child self-reports and parent proxy-reports, indicating excellent internal consistency.

#### 2.4.2. Explanatory variables

The background information about the study participants included age, sex, grade, weight, height, BAZ, nutritional status, perceived household economic status, and fathers' and mothers' highest education levels.

Children's age was divided into three groups, including the 8, 9 and 10 year-old groups. Similarly, they were grouped into grades they were attending, namely grade 3, grade 4 and grade 5.

The children were classified as obese if (BAZ > +2SD) and normal weight if:

−2SD≤BAZ≤+1SD

Perceived household economy (self assessed by parents) included three levels: High income, middle income, and low income.

Parents' education attainment was categorized into three levels, namely high school or lower, undergraduate (including secondary technical school, college, university), and post graduate.

### 2.5. Data analysis

Anthropometric data were analyzed with Anthro plus 2007. Demographic and QOL data were entered using Epidata 3.0. The data were then analyzed with Stata 13.1. Descriptive analyses, including means and SD were calculated, and independent T-test and Chi-square test were used to compare QOL scores between groups with a *p*-value < 0.05 indicating a significant result. Bivariate analyses and multivariable linear regression were employed to identify factors affecting children's QOL. Independent variables involved in bivariate analyses includes children's age and sex and nutritional status, householde economic and parent's education level. Multivariable linear regression models were constructed which contained those variables significant in unadjusted analyses at the alpha = 0.05 level, and were additionally adjusted for age and gender.

### 2.6. Ethical considerations

The study was approved by the Ethical Committee of Hanoi University of Public Health. The research team obtained approval to conduct the study from the Boards of Management of the two primary schools before data collection was undertaken. The study was conducted only when both parents' and their children's consent was obtained. The research staff explained clearly to the students and their parents about the study objectives and the study procedures and informed them that participation in the study was completely voluntary, which means that they could withdraw from the study at any time without bearing any consequences.

## 3. Results

Socio-demographic and weight-related characteristics of the sample are presented in Table 1. The results show that the prevalence of obesity in male and female children was quite similar (32.8% and 34.9%, *p* = 0.759). More than half of the parents completed secondary technical school, college and university education, and 30% had post-graduate education. Ninety per cent of parents reported they were from middle-income families, while only 7.6% and 2.4% reported being from high and low family income, respectively.

**Table 1.** Study participant's sociodemographic characteristics.

	Male (n = 201) (n, %)	Female (n = 63) (n, %)	Total (n = 264) (n, %)	*p*
*Age*				
8	78 (38.8)	28 (44.4)	106 (40.2)	0.409
9	76 (37.8)	18 (28.6)	94 (35.6)	
10	47 (23.4)	17 (27)	64 (24.2)	
*Nutritional status*				
Obesity	66 (32.8)	22 (34.9)	88 (33.3)	0.759
Normal weight	135 (67.2)	41 (65.1)	176 (66.7)	
*Parental education* (reported by parents)				
High school or lower	23 (11.6)	7 (11.3)	30 (11.5)	0.696
Graduate	119 (60.1)	34 (54.8)	153 (58.8)	
Post-graduate	56 (28.3)	21 (33.9)	77 (29.6)	
*Household economy* (reported by parents)				
Rich	16 (8.4)	3 (5.2)	19 (7.6)	0.27
Middle-income	169 (88.5)	55 (94.8)	224 (90.0)	
Poor	6 (3.1)	0 (0)	6 (2.4)	

Table 2 shows the mean scores for QOL overall scales and subscales by nutritional status. The results show that the self-report and parent-proxy mean scores for the overall scale for obese children were statistically significantly lower than those for normal-weight ones (*p* < 0.001). Obese children's self-reported total QOL mean score was 77.6; 7 points lower than that of their normal weight counterparts'.

**Table 2.** Parent- and Child-reported PedsQL total and subscale scores by normal and obese children.

	Parent-Proxy			Child Self-report		
	Normal Mean (SD) (n = 176)	Obese Mean (SD) n = 88	*p*	Normal Mean (SD) (n = 176)	Obese Mean (SD) n = 88	*p*
Total	84 (10.4)	80.7 (13.1)	0.027	84.6 (12.6)	77.6* (15.5)	0.0001
Physical functioning	86 (12.9)	84.9 (13.8)	0.542	86.5 (13.3)	81.5* (15.2)	0.005
Emotional functioning	79.5 (16.6)	73.1 (22)	0.009	78.7 (17.5)	71.6*(21.3)	0.005
Social functioning	88.1 (14.5)	84.4 (16.6)	0.068	88.6 (14.2)	81.6* (18.6)	0.001
School functioning	82.6 (15.8)	80.3 (16.3)	0.289	84.5 (15.6)	75.9* (18.9)	0.001

Note: * *p* < 0,001 vs. obese of parent-proxy, t-test.

With regards to the subscales, almost all parent-proxy PedsQL scores surpassed child self-reported ones in both obese and normal weight groups (*p* < 0.001). For all four subscales, child self-reported PedsQL mean scores for the obese group were statistically lower than those for the normal weight group. Emotional functioning score was the lowest among the four PedsQL subscales (71.6). Although parent-proxy PedsQL mean scores for the obese group were also inferior to those for the normal weight group in all of the four subscales, evidence was insufficient to draw a conclusion about the statistically significant differences in the physical, social and school functioning between the two groups of children (*p* > 0.05).

Table 3 demonstrates PedsQL scores by quartiles. Based on child self-report results, 79% of normal weight children had a good total QOL (highest quartile). This proportion was significant than that among obese children (56.8%) (*p* = 0.01). Similarly, the proportions of normal weight children and obese children with good QOL reported by parents were 79.5% and 65.9%, respectively (*p* < 0.05). Regarding subscale scores, obese children's emotional functioning, whether reported by parents or children themselves, significantly differed from that of normal weight counterparts. The same pattern was observed for emotional functioning with the prevalence of good emotional functioning among obese chidlren and normal weight children data being 46.6% and 54.4% (*p* < 0.05), respectively in self-reports and 47.7% and 53.4%, respectively in proxy-reports (*p* < 0.05). Besides, 2.3% of obese children classifying their emotional functioning as poor (scores beloing to the lowest quartile) while no normal weight children reported poor emotional functioning.

**Table 3.** Quality of life based on quartiles of PedsQL scores.

	Parent-Proxy					Child Self-report				
	Normal		Obese		*p*	Normal		Obese		*p*
	n	%	n	%		n	%	n	%	
*Total scales*										
≥75%	140	79.5	58	65.9	0.017	139	79.0	50	56.8	0.001
50–75%	36	20.5	29	33.0		34	19.3	35	39.8	
25– ≤ 50%	0	0.0	1	1.1		3	1.7	3	3.4	
≤25%	0	0	0	0		0	0	0	0	
*Physical functioning*										
≥75%	123	69.9	60	68.2	0.411	132	75.0	58	65.9	0.225
50–75%	50	28.4	24	27.3		37	21.0	27	30.7	
25– ≤ 50%	3	1.7	4	4.5		7	4.0	3	3.4	
≤25%	0	0	0	0		0	0	0	0	
*Emotional functioning*										
≥75%	94	53.4	42	47.7	0.048	96	54.5	41	46.6	0.018
50–75%	65	36.9	31	35.2		58	33.0	32	36.4	
25– ≤ 50%	17	9.7	12	13.6		22	12.5	13	14.8	
≤25%	0	0.0	3	3.4		0	0.0	2	2.3	
*Social functioning*										
≥75%	124	70.5	59	67.0	0.814	96	54.5	41	46.6	0.179
50–75%	46	26.1	25	28.4		58	33.0	32	36.4	
25– ≤ 50%	6	3.4	4	4.5		22	12.5	13	14.8	
≤25%	0	0	0	0		0	0.0	2	2.3	
*School functioning*										
≥75%	109	61.9	47	53.4	0.331	121	68.8	47	53.4	0.041
50–75%	60	34.1	35	39.8		43	24.4	32	36.4	
25– ≤ 50%	7	4.0	6	6.8		12	6.8	8	9.1	
≤25%	0	0	0	0		0	0.0	1	1.1	

**Figure 1.**
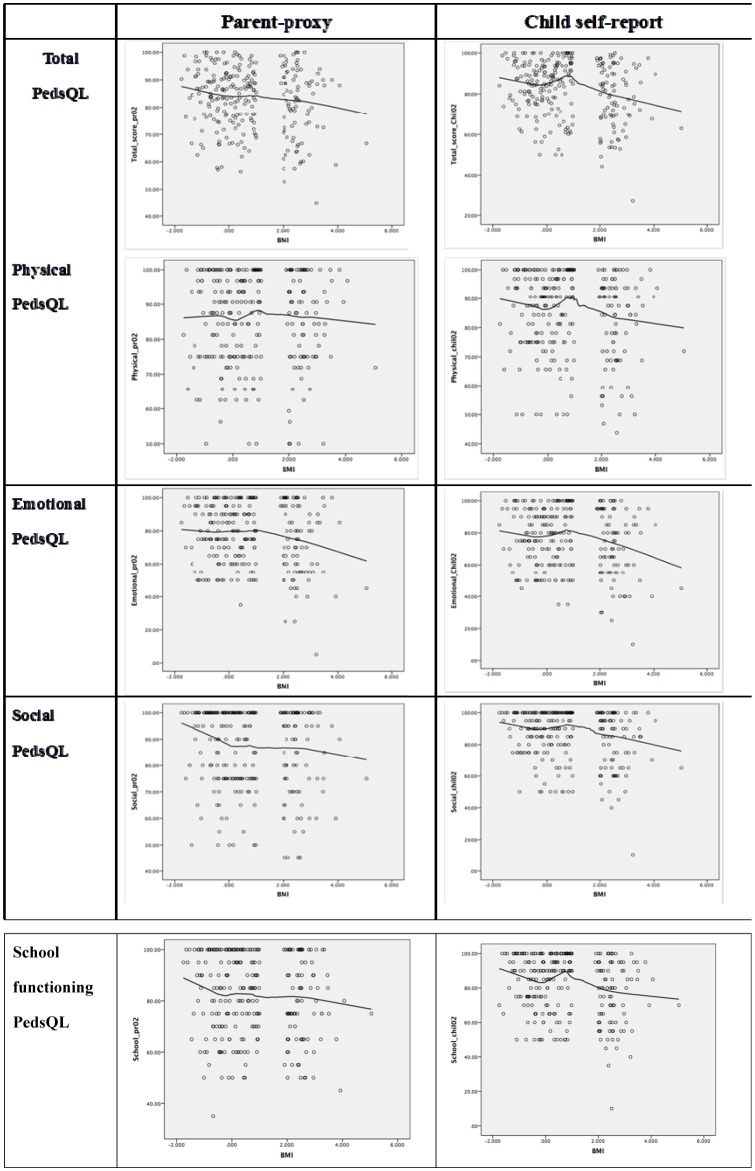
PedsQL scale scores reported by parent-proxies and children.

Figure 1 presents the distribution of PedsQL scores by BAZ. From parents' reports, the higher the obese children's BAZ, the lower the total PedsQL score. However, the child self-reported total PedsQL score had a different pattern where scores increased as BAZ increased within the range from 0 and 2, but gradually decreased as BAZ increased for children having BAZ equal or greater than 2.

The lines representing physical and school functioning QOL in Figure 1 show that the child self-report and parent-proxy PedsQL scores increased as BAZ increased in children with BAZ between 0 and 2, but gradually declined as BAZ increased in obese children with BAZ equal or greater than 2. Child self-reported PedsQL score among children with BAZ equal or greater than 2 saw a more obvious reducing trend than did the parent-proxy PedsQL score.

The parent-proxy and child self-reported emotional and social scores had a similar pattern. This shows that among those obese children who have BAZ equal or greater than 2 or among obese children, increased BAZ is associated with the decreased emotional and social functioning.

Table 4 demonstrates univariate and multivarable linear regression analysis of correlates of QOL. In bivariate analysis, PedsQL scores (both by parent proxy and child self report) were significantly different from the nutritional status (obesity and normal weigh) and by age group *p* < 0.05. In multivariable analysis, the results from parent proxy showed that obese children had a 3.08 point lower PedsQL score than normal weight children (*p* < 0.05). Meanwhile, based on child self-report, the mean PedsQL score of obese children was 6.38 points lower than that of their normal weight peers (*p* < 0.01). QOL remained different by age of the children in multivariable regression analysis. One year increase in age was associated with 1.96 score increase in QOL by perent proxy and 2.58 by child self report (*p* < 0.05).

**Table 4.** Bivariate and multivariable linear regression analysis of correlates of QOL.

	Bivariate analysis			Multivariable analysis		
	Coeff	95% CI	*p* value	Coeff	95% CI	*p* value
*Parent's proxy*						
*Nutritional status*						
Normal weigh	1					
Obesity	−3.31	(−6.24; −0.38)	0.027	−3.08	(−6.14; −0.04)	0.047
*Sex*						
Female	1					
Male	−0.72	(−3.99; 2.55)	0.665	−4.02	(−3.84; 2.99)	0.807
*Age*						
8	1			1.96	(0.14; 3.79)	0.035
9	3.20	(0.04; 6.36)	0.047			
10	1.69	(−0.19; 3.7)	0.077			
*Household economic*						
Rich	1					
Middle-income	−3.91	(−13.43; 5.60)	0.418			
Poor	−1.73	(−6.93; −3.48)	0.499			
*Parental education*						
High school or lower	1					
Graduate	0.11	(−4.37; 4.58)	0.963			
Post-graduate	0.75	(−1.82; 3.33)	0.564			
*Child self report*						
*Nutritional status*						
Normal weigh	1					
Obesity	−6.94	(−10.44; −3.43)	0.05	−6.38	(−10.02; −2.76)	0.001
*Sex*						
Female	1					
Male	−0.44	(−4.44; −3.55)	0.827	0.08	(−3.973; −4.135)	0.969
*Age*						
8	1			2.58	(0.68; 5.02)	0.014
9	−0.44	(−4.44; 3.55)	0.827			
10	5.31	(0.90;9.72)	0.019			
*Household economic*						
Rich	1					
Midle income	−7.76	(−19.27; 3.7)	0.185			
Poor	−8.38	(−20.76; 3.99)	0.175			
*Parental education*						
High school or lower	1					
Graduate	0.13	(−5.4; 5.668)	0.962			
Post-graduate	3.2	(−2.78; 9.19)	0.292			

## 4. Discussion

Obesity is an alarming public health problem in children and acts as an early risk factor for various diseases and mortality in adults. Childhood obesity is likely to remain into adulthood, and therefore, is the early onset of certain chronic diseases that last a lifetime. Obesity leads to different consequences related to QOL, resulting in poorer physical, social, psychological and school functioning [9]–[12].

In our study, QOL was measured by the PedsQL 4.0 generic score scales. Both parents and children reported statistically significantly lower PedsQL scores for obese children as compared to that for normal weight children. Child self-reported PedsQL scores in terms of physical, emotional, social and school functioning suggest that obesity can have negative effects on children's daily life. These findings are consistent with those from previous research conducted in the UK, Egypt and Malaysia in which obese children reported lower QOL scores in all subscales of the PedsQL compared to normal weight ones [11],[17],[19].

In our study, the child self-reported physical PedsQL scores in obese children were significantly lower than those in normal weight children while parent-proxy reports did not show a significant difference between the two groups (Table 2). A possible explanation is that excess body weight may hinder children from being involved in physical activity, while their parents often prefer them to be “plump”. As a result, parents often fail to notice the difference between their children and others with normal weight. The proportions of obese children and their parents reporting high physical scores (in the last quartile, ≥75%) were 65.9% and 68.2%, respectively while the percentages for normal weight children were 75% and 69.9%, respectively (*p* > 0.05) (Table 3). These results support previous research that failed to find different level of physical functioning between obese and normal weight children [11],[14],[19],[20].

Parent-proxy and child self-reported emotional PedsQL scores in our study were the lowest in all four PedsQL subscales (Table 2). Only 46.6% of obese children reported good emotional scores (in the highest quartile, ≥75%), as opposed to 54.4% of their normal weight peers (*p* < 0.05) (Table 3). Besides, emotional and school functioning began to show a clearly decreasing trend as BAZ increased from 2 (which is the threshold for obesity classification) (Figure 1). This indicates the emotional functioning among obese children in our study was poorer than that among their normal weight peers. This result was consistent with those from research in Germany, the UK, Malaysia and the USA [11],[17],[21],[22]. It can be explained by the impacts of obesity upon children related to cultural and social pressure in their communities, stigma, teasing and bullying directed towards heavy children; all these badly affect children's emotions. The poor emotional functioning found among obese children also suggests that all psycho-social aspects of obesity are still neglected in interventions aiming to improve the health status of obese children [23]. Therefore, subsequent interventions for obese children in Vietnam need to provide comprehensive solutions, covering both physical and psycho-social aspects. Furthermore, with the increase in obesity prevalence in children, parents, teachers, friends and society need to recognize psycho-social impacts of obesity on children in supporting those with heavy weight.

In our study, child self reports revealed that the school functioning PedsQL score in obese children were far lower than that in normal weight children (*p* < 0.05). This result supports findings of a previous a study in Hong Kong [10]. The Hong Kong study found that normal weight children had a considerably higher school functioning PedsQL score than obese ones. However, in our study, parent-proxy reports did not show significant difference in school functioning between the two groups. This result is consistent with findings of previous studies in Australia and Malaysia. These two studies showed that both groups had similar school functioning scores through parent-proxy reports [11],[14].

In this study, we investigated the impacts of obesity on the QOL, controlling for potential confounding factors including age, sex, parental education and household economy in multivariable analysis (Table 4). The results again confirmed that the PedsQL scores of obese children were lower than those of normal weight children. Results also showed that the older the children, the higher the QOL score. These results were consistent with those from previous research [13],[14],[24],[25]. This result can be explained by that in very young children, there are very little or no differences in PedsQL scores stratified by age and sex. However, those differences emerge or increase during adolescence, when children become more aware of their body image [9],[26],[27].

The study result showed that PedsQL scores based on parent-proxy reports, including both the overall scores and subscale ones were significantly higher than those from self-reports (Table 2). This result is consistent with that reported by Marie Buttitta et al. in a recent systematic review. In the latter study, the authors reviewed 34 articles on obese children's QOL, 19 of which used the PedsQL questionnaire. Five out of seven studies that compared between self-reports andparent reports found that QOL scores were significantly lower when reported by parent than by children themselves for some or all of the QOL dimensions [5]. As mentioned above, since parents tended to prefer their children to have “chubby bodies”, they might ignore or underestimate negative signs of functioning associated with heavry weight, hence report better QOL in obese children. In other words, the children's QOL reported by their parents was often higher than that based on self-reports. As parents can play important roles in deciding health care for their children, the study results imply the need for raising awareness of Vietnamese parents about obesity and its consequences on children.

Our study has limitations related to its sample and the survey instrument used to assess QOL. Our study was conducted on students at two schools (one public and one private) in an urban district of Hanoi; therefore, the study results possibly cannot be applied to children and adolescents in other communities, such as those in rural areas. Besides, the PedsQL 4.0 used in this study is an instrument for assessing the general QOL and it contains no questions designed specifically for obese children. Therefore, further research is needed to analyze more accurate impacts of obesity on children's QOL.

## 5. Conclusions and recommendations

This study suggests that obesity has negative effects on children's QOL. Obese children reported having lower PedsQL scores than their normal weight peers', in total PedsQL and all four subscales of physical, emotional, social and school functioning, and especially lowest in emotional functioning.

The study results suggest contribute to raising awareness about consequences of obesity among Vietnamese children, emphasing its negative impact on psycho-social functioning. The study findings indicate the needs for more comprehensive policies and invention programs for preventing obesity and lessening its impact on Vietnamese children's health. The current policies and interventions should extend their scope from only focusing on dietary management and increasing physical activities to a more holistic approach, involving also activities that promote social and psychological well-being. In addition, the PedsQL is a simple, easy-to-use and reliable instrument for assessing health-related QOL. It has the potential to become a useful intrument for research in children's QOL as well as for evaluation of intervention programs aimed to increase QOL among children in Vietnam.
